# The CMS19 disease model specifies a pivotal role for collagen XIII in bone homeostasis

**DOI:** 10.1038/s41598-022-09653-4

**Published:** 2022-04-07

**Authors:** A. V. Kemppainen, M. A. Finnilä, A. Heikkinen, H. Härönen, V. Izzi, S. Kauppinen, S. Saarakkala, T. Pihlajaniemi, J. Koivunen

**Affiliations:** 1 ECM-Hypoxia Research Unit, Faculty of Biochemistry and Molecular Medicine, University of Oulu, P.O. Box 5400, 90014 Oulu, Finland; 2 Research Unit of Medical Imaging, Physics and Technology, Faculty of Medicine, University of Oulu, P.O. Box 5000, 90014 Oulu, Finland; 3 Faculty of Medicine, University of Oulu, 90014 Oulu, Finland; 4 Foundation for the Finnish Cancer Institute, Tukholmankatu 8, 00130 Helsinki, Finland; 5 Department of Diagnostic Radiology, Oulu University Hospital, Oulu, Finland

**Keywords:** Bone development, Bone remodelling, Bone, Ageing, Developmental disorders

## Abstract

Mutations in the *COL13A1* gene result in congenital myasthenic syndrome type 19 (CMS19), a disease of neuromuscular synapses and including various skeletal manifestations, particularly facial dysmorphisms. The phenotypic consequences in *Col13a1* null mice (*Col13a1*^*−/−*^) recapitulate the muscle findings of the CMS19 patients. Collagen XIII (ColXIII) is exists as two forms, a transmembrane protein and a soluble molecule. While the *Col13a1*^*−/−*^ mice have poorly formed neuromuscular junctions, the prevention of shedding of the ColXIII ectodomain in the *Col13a1*^*tm/tm*^ mice results in acetylcholine receptor clusters of increased size and complexity. In view of the bone abnormalities in CMS19, we here studied the tubular and calvarial bone morphology of the *Col13a1*^*−/−*^ mice. We discovered several craniofacial malformations, albeit less pronounced ones than in the human disease, and a reduction of cortical bone mass in aged mice. In the *Col13a1*^*tm/tm*^ mice, where ColXIII is synthesized but the ectodomain shedding is prevented due to a mutation in a protease recognition sequence, the cortical bone mass decreased as well with age and the cephalometric analyses revealed significant craniofacial abnormalities but no clear phenotypical pattern. To conclude, our data indicates an intrinsic role for ColXIII, particularly the soluble form, in the upkeep of bone with aging and suggests the possibility of previously undiscovered bone pathologies in patients with CMS19.

## Introduction

Collagens are the central structural constituent of the extracellular matrix (ECM). In addition to their structural role, collagens regulate cell signaling via cell surface receptors such as integrins. Altogether 28 different collagen types have been identified, representing a large variety of structures, expression profiles and binding partners. The large family of collagens has been implicated in various genetic and acquired human disorders^1–3^.

Collagen XIII (ColXIII) is a membrane-associated collagen, comprised of a short N-terminal cytosolic domain, a transmembrane domain and a largely collagenous ectodomain^4,5^. The ectodomain interacts with several ECM components such as fibronectin, nidogen-2 and perlecan^6^ as well as α1ß1^7^ and α11ß1 integrins^8^. The ectodomain of ColXIII can be cleaved from the cell membrane by furin proteases, forming a soluble biologically active variant of ColXIII^9,10^. ColXIII is expressed in various mesenchymal cell lines and tissues during development, being mostly associated with basement membrane and junctional structures where it exists in adult tissue^4,11^. A distinct high expression has been demonstrated in postnatal neuromuscular junctions (NMJs) and periosteal and endosteal osteoblasts^12–15^. Recently, ColXIII was shown to be a basement membrane component in the hair germ–dermal papilla interface^16^.

The α-chain of the homotrimeric ColXIII protein is encoded by the *COL13A1* gene^4^. Different loss-of-function mutations of *COL13A1* manifest clinically as congenital myasthenic syndrome type 19 (CMS19)^17–21^. In CMSs, mutations of NMJ-affiliated proteins and the subsequently abnormal function of NMJs manifest physiologically as a distinct spectrum of symptoms, which often includes muscle weakness, respiratory difficulties, and ptosis^22^. Similarly to CMS19 patients, ColXIII null mice (*Col13a1*^*−/−*^) show impaired NMJ development and function as well as reduced muscle strength^13,23^. The mouse line with a mutation in the furin-type proprotease recognition sequence of ColXIII (*Col13a1*^*tm/tm*^) lacks only the shed form of ColXIII. Neuromuscular synapses in the muscle tissue of *Col13a1*^*tm/tm*^ mice exceed their typical physiological complexity^23^. In addition to the characteristic symptoms, CMS19 patients present with varying skeletal abnormalities. These include thoracolumbar scoliosis, kyphosis, and dysmorphic facial features such as an elongated face, micrognathia, and high-arched palate^17,19,20^.

The fundamental pathophysiological properties of CMS19 are closely replicated in the *Col13a1*^*−/−*^ mice^23^. Moreover, pre- and postsynaptic NMJ defects have been discovered in mutated ColXIII mouse lines (*Col13a1*^*−/−*^*, Col13a1*^*oe*^*,* and *Col13a1*^*tm/tm*^)^13,15,23^. While the NMJ development and the neuromuscular properties of the *Col13a1*^*−/−*^ and the *Col13a1*^*tm/tm*^ mice have been studied, their skeletal characteristics are completely unknown despite the fact that human CMS19 patients demonstrate skeletal phenotypes. In this study, we analyzed the bone tissue of *Col13a1*^*−/−*^ and *Col13a1*^*tm/tm*^ mice and compared our findings to those reported in the human CMS19 patients.

## Results

### Diaphyseal cortical bone area and thickness are reduced in aged *Col13a1*^*−/−*^ and *Col13a1*^*tm/tm*^ mice

**Figure 1:**
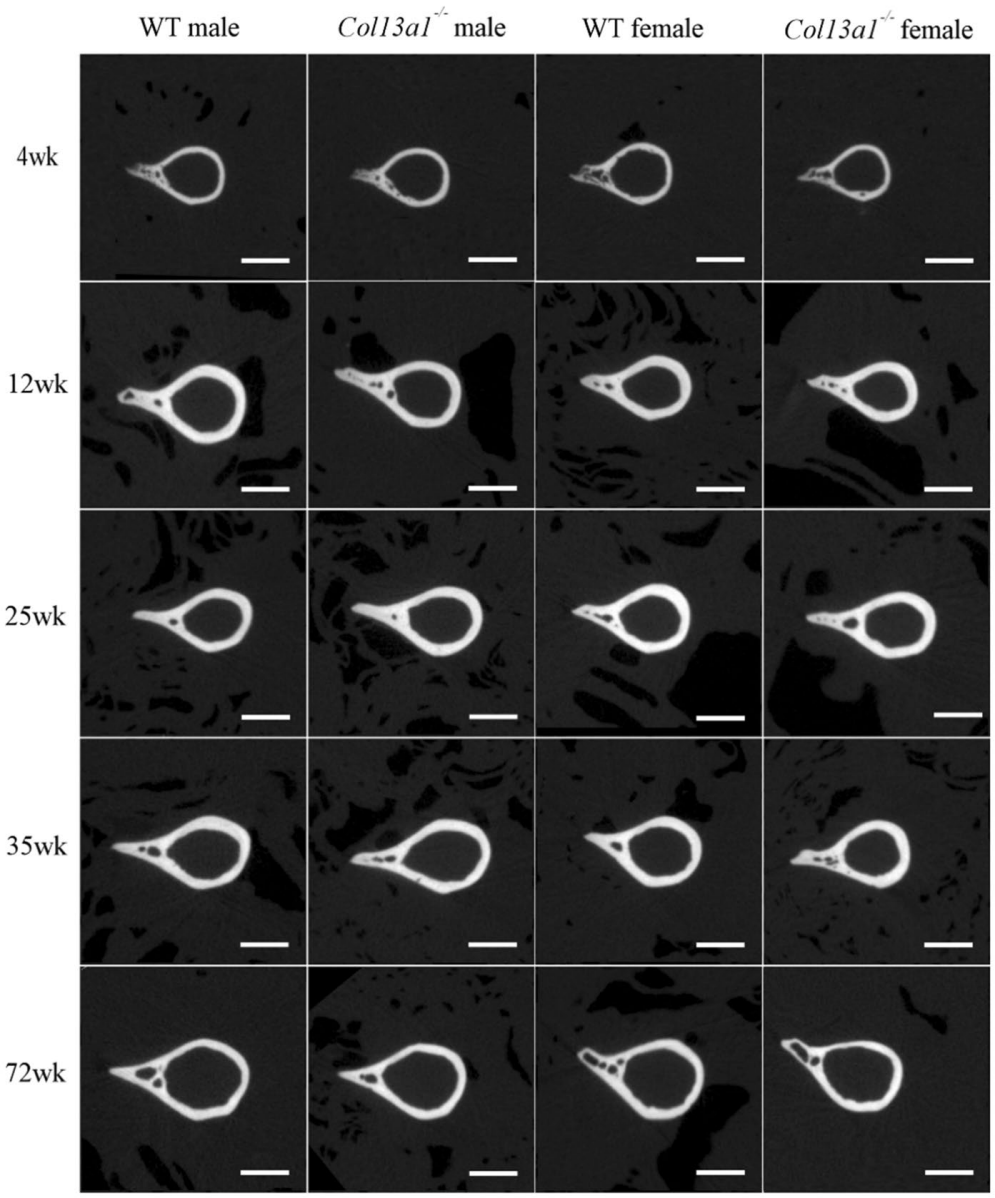
Single scaled mid-diaphyseal axial tomographic µCT images of male and female *Col13a1*^*−/−*^ mouse femurs and respective controls at the 4-, 12-, 25-, 35, and 72-week time points. Scale bars 1 mm.

**Figure 2:**
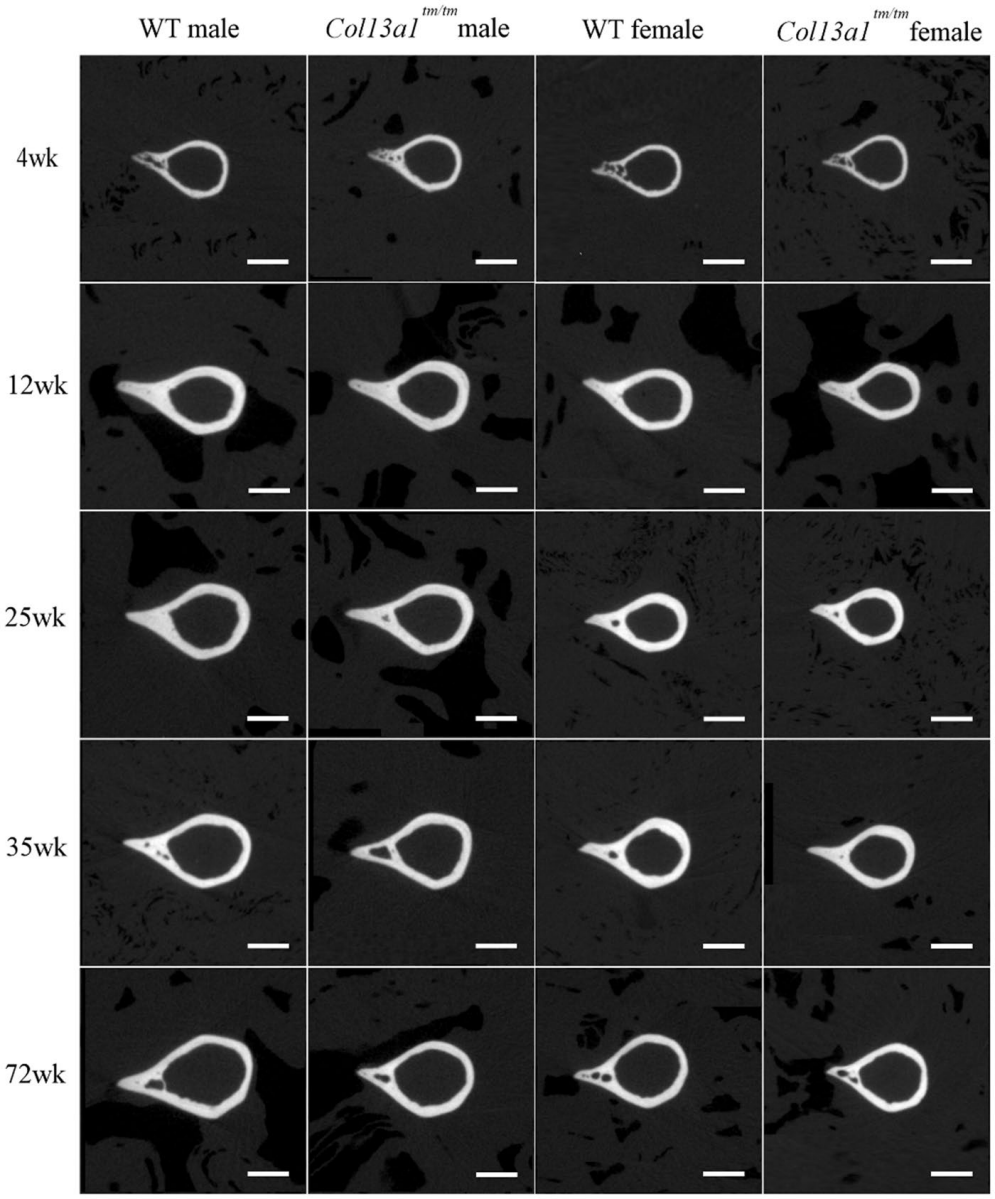
Single-scaled mid-diaphyseal axial tomographic µCT images of male and female *Col13a1*^*tm/tm*^ mouse femurs and respective controls. From the 4- to 35-week time points, diaphyseal cortical bone morphology remained mostly unaltered in the *Col13a1*^*tm/tm*^ samples compared to the controls, whereas at 72 weeks a slight reduction in bone mass could be seen compared to the WT samples. Scale bars 1 mm.

**Figure 3:**
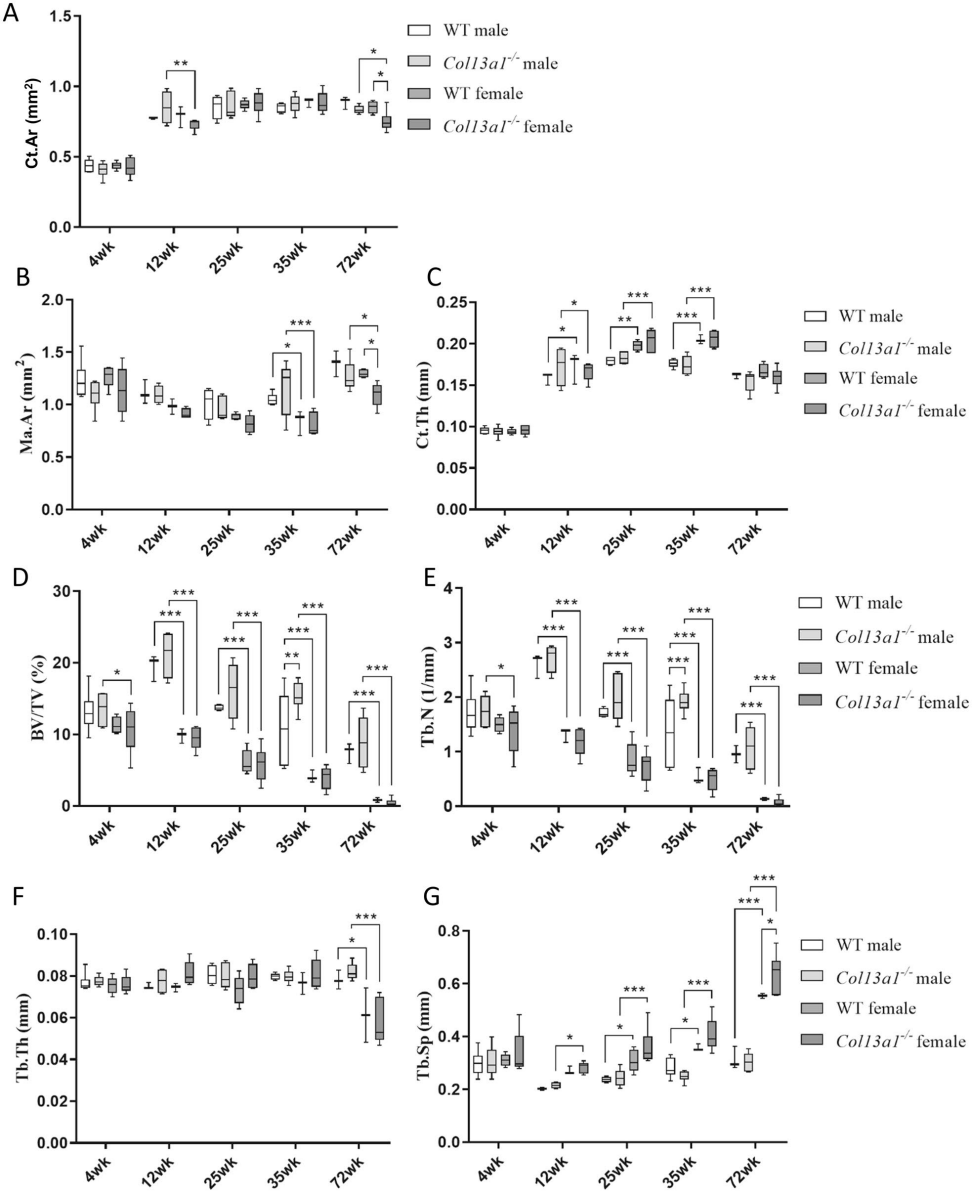
Summary of morphometric parameters of diaphyseal cortical bone and distal femoral trabecular bone of male and female *Col13a1*^*−/−*^ mice and WT controls. Cortical area (Ct.Ar, **A**) and medullary area (Ma.Ar, **B**) were significantly reduced in the female *Col13a1*^*−/−*^ mice at the 72-week compared to the controls. There was no differences in cortical thickness (Ct.Th., **C**) between genotypes. Bone volume fraction (BV/TV, **D**) and trabecular number (Tb.N, **E**) were higher in 35-week-old *Col13a1*^*−/−*^ males compared to WT. There was no differences in trabucular thickness (Tb.Th., **F**) between genotypes. Trabecular separation (Tb.Sp, **G**) was higher in 72-week-old *Col13a1*^*−/−*^ females compared to WT. n(WT male): 3–6; n(*Col13a1*^*−/−*^ male): 4–7; n(WT female): 3–5; n(*Col13a1*^*−/−*^ female): 5–6; the whiskers represent min to max; *q < 0.05, **q < 0.01, ***q < 0.001 determined by two-way ANOVA and followed by the false discovery rate.

**Figure 4:**
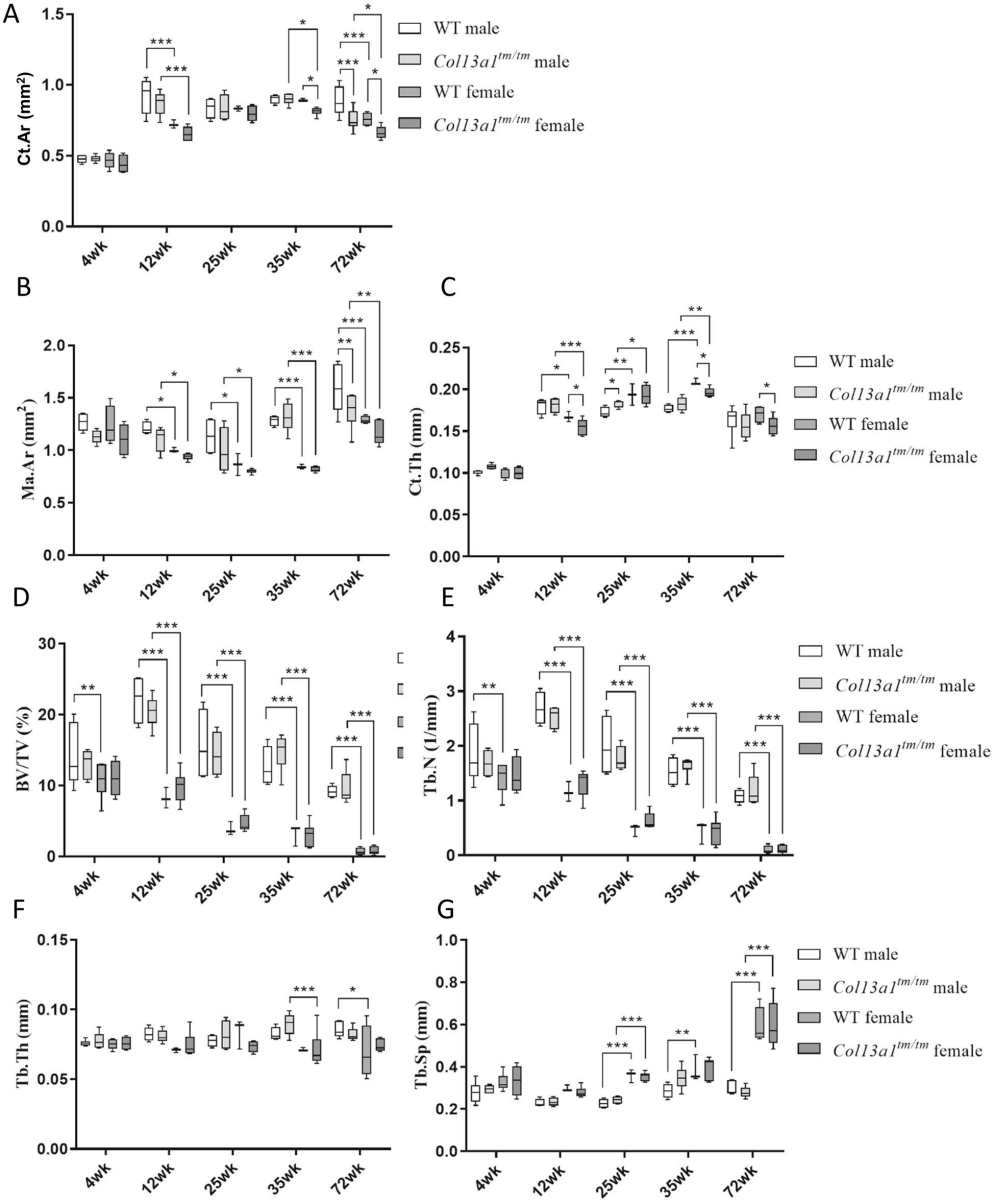
Summary of morphometric parameters of diaphyseal cortical bone and distal femoral trabecular bone of male and female *Col13a1*^*tm/tm*^ mice and WT controls. The cortical bone area (Ct.Ar, **A**) was significantly reduced in the female *Col13a1*^*tm/tm*^ mice at the 35- and 72-week and in males at the 72-week. The medullary area (Ma.Ar, **B**) was significantly lower in *Col13a1*^*−/−*^ mice at the age of 72-week compared to the controls The cortical thickness (Ct.Th, **C**) was reduced in the female *Col13a1*^*tm/tm*^ mice at 12-, 35- and 72-week-old mice. The trabecular bone was unaltered between *Col13a1*^*tm/tm*^ and WT samples (**D**–**G**). n(WT male): 4–6; n(*Col13a1*^*tm/tm*^ male): 4–6; n(WT female): 2–6; n(*Col13a1*^*tm/tm*^ female): 4–6; the whiskers represent min to max; *q < 0.05, **q < 0.01, ***q < 0.001 determined by two-way ANOVA and followed by the false discovery rate.

To determine the effects of absence of ColXIII in tubular bone morphometry, femurs of male and female *Col13a1*^*−/−*^ and *Col13a1*^*tm/tm*^ mice were imaged using µCT. Some reduction of bone mass could be depicted in the 72-week-old female *Col13a1*^*−/−*^ and *Col13a1*^*tm/tm*^ samples compared to their respective WT control samples in single mid-diaphyseal axial images (Figs. 1 and 2). The 2D morphometric analysis showed a significant reduction of the cortical bone and the medullar area in the female *Col13a1*^*−/−*^ mice at the 72-week time point (Fig. 3A,B). In the *Col13a1*^*tm/tm*^ mice, the cortical bone area and cortical thickness were reduced in the 35-week-old as well as 72-week-old female mice (Fig. 4A,C). Additionally, the cortical bone and the medullar areas were reduced in the 72-week-old male *Col13a1*^*tm/tm*^ mice compared to the respective WT controls (Fig. 4A,B).

3D-morphometric analysis of distal femoral trabecular bone did not show major alterations in trabecular architecture or volume in the *Col13a1*^*−/−*^ or the *Col13a1*^*tm/tm*^ mice compared to the respective WT control samples (Figs. 3D–G, 4D–G). The differences in the trabecular architecture between sexes and phenotypes were quite evident in the 3D reconstruction images of distal femoral trabecular bone and are consistent with the 3D analysis (Supplementary Figs. S1 and S2, Figs. 3D–G, 4D–G). Additionally, the femoral length was measured from each sample, but no significant differences were observed in the *Col13a1*^*−/−*^ or the *Col13a1*^*tm/tm*^ mice in either gender at any time point compared to respective WT controls (Supplementary Fig. S3).

### Cortical bone area and thickness were reduced in the metaphyseal region in *Col13a1*^*−/−*^ and *Col13a1*^*tm/tm*^ mice

**Figure 5:**
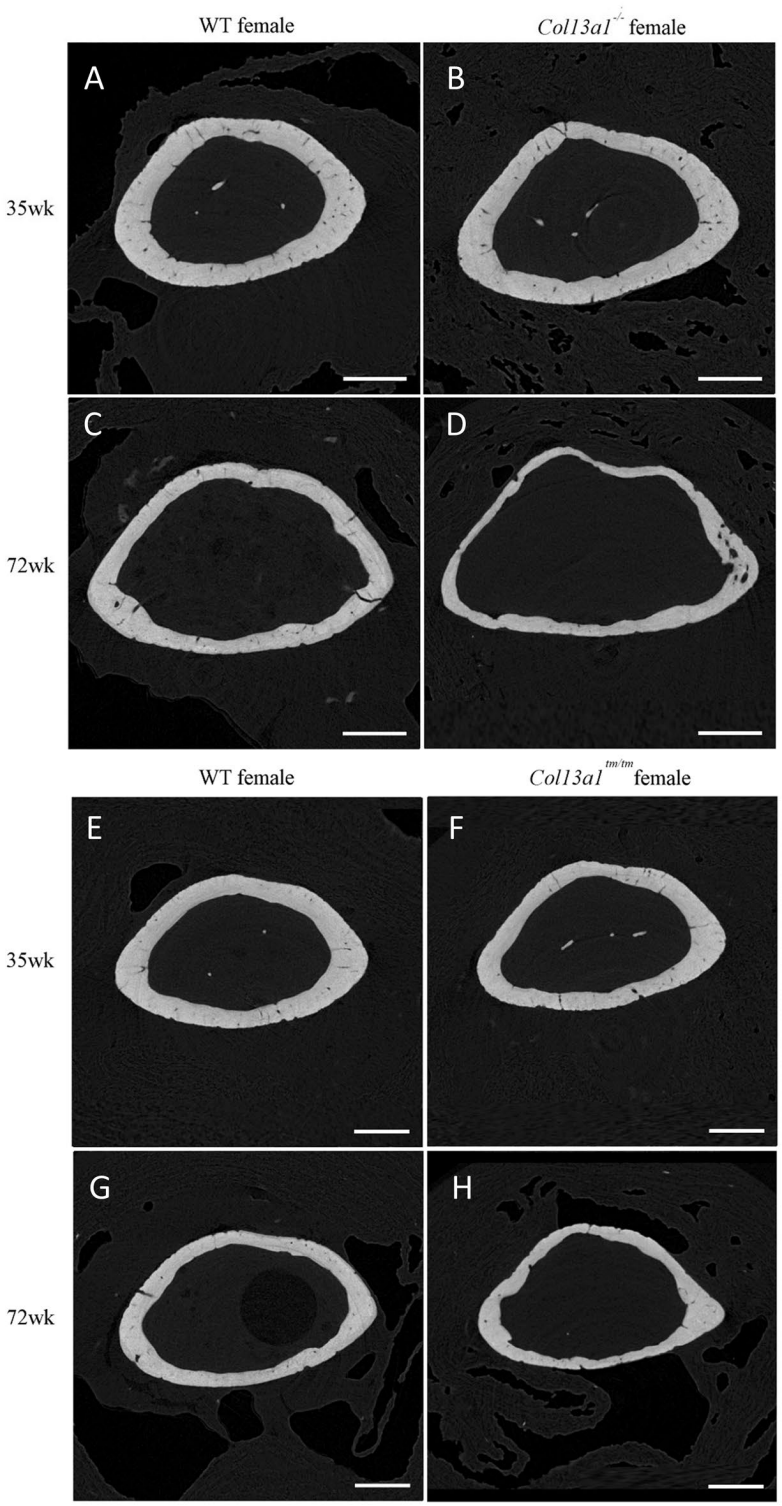
Single scaled axial tomographic µCT images of distal metaphyseal femoral cortical bone in female *Col13a1*^*−/−*^,* Col13a1*^*tm/tm*^ and WT control mice at the age of 35 and 72 weeks. Whereas bone morphology was similar at the 35-week time point (**A**,**B**), cortical bone mass was clearly reduced in the *Col13a1*^*−/−*^ mice at the 72-week time point compared to the WT samples (**C**,**D**). The metaphyseal cortical bone mass was significantly reduced in the *Col13a1*^*tm/tm*^ mice at both time points compared to the WT controls (**E**–**H**). Scale bars 500 µm.

**Figure 6:**
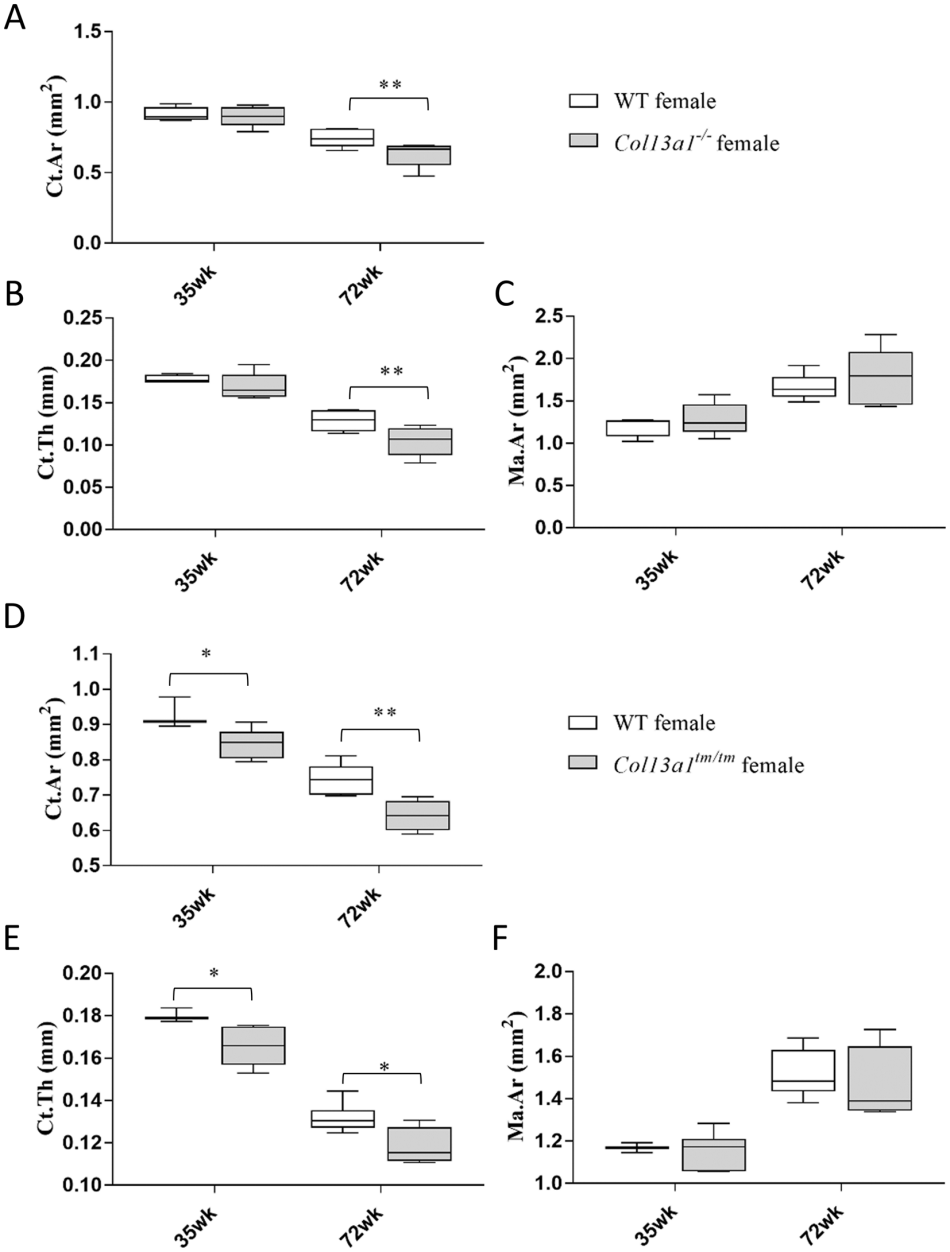
Morphometric parameters for HRCT analysis of metaphyseal cortical bone. The *Col13a1*^*−/−*^ female mice and respective WT controls (**A**–**C**). There were no statistically significant differences in the cortical area (Ct.Ar, **A**) or thickness (Ct.Th, **B**) or the medullary area (Ma.Ar, **C**) at the 35-week time point. At the 72-week time point, Ct.Ar and Ct.Th were significantly reduced in the *Col13a1*^*−/−*^ samples compared to the WT mice. The *Col13a1*^*tm/tm*^ female mice and respective WT controls (**D**–**F**). The cortical area (Ct.Ar, **D**) and thickness (Ct.Th, **E**) were significantly reduced in the *Col13a1*^*tm/tm*^ mice at the 35- and 72-week time points compared to the respective controls. The medullary area (Ma.Ar, **F**) was unaltered. n(WT female): 4–5; n(*Col13a1*^*−/−*^ female): 5; n(WT female): 3–6; n(*Col13a1*^*tm/tm*^ female): 4–6; the whiskers represent min to max; **q < 0.01 determined by two-way ANOVA and followed by the false discovery rate.

To confirm our findings in the *Col13a1*^*−/−*^ and the *Col13a1*^*tm/tm*^ mice, a high-resolution µCT scanner was utilized to re-analyse the 35- and 72-week-old female *Col13a1*^*−/−*^ and *Col13a1*^*tm/tm*^ mouse femur samples (Fig. 5). Scanner limitations enabled imaging only the distal third of the diaphysis, the metaphysis, and the distal growth plate to fit into a single image acquisition sequence. Thus, cortical bone was analyzed from the metaphyseal region. The cortical bone area and thickness were comparable to controls at the 35-week time point but significantly decreased in the 72-week-old *Col13a1*^*−/−*^ female mice (Fig. 6A,B) and in the 35- and 72-week-old *Col13a1*^*tm/tm*^ mice (Fig. 6D,E) while the medullary area was unaltered in both genotypes (Fig. 6C,F). In the 72-week-old female *Col13a1*^*−/−*^ mice, the distal metaphyseal cortical bone area was 16% less and the cortical thickness 19% less compared to WT (Fig. 6A,B). In the female *Col13a1*^*tm/tm*^ mice, the distal metaphyseal cortical bone area was 9% less and the cortical thickness 8% less at the 35-week time point and 14% and 10% less, respectively, at the 72-week time point compared to WT controls (Fig. 6D,E).

### Cephalometric analysis of *Col13a1*^*−/−*^ and *Col13a1*^*tm/tm*^ mice

**Figure 7:**
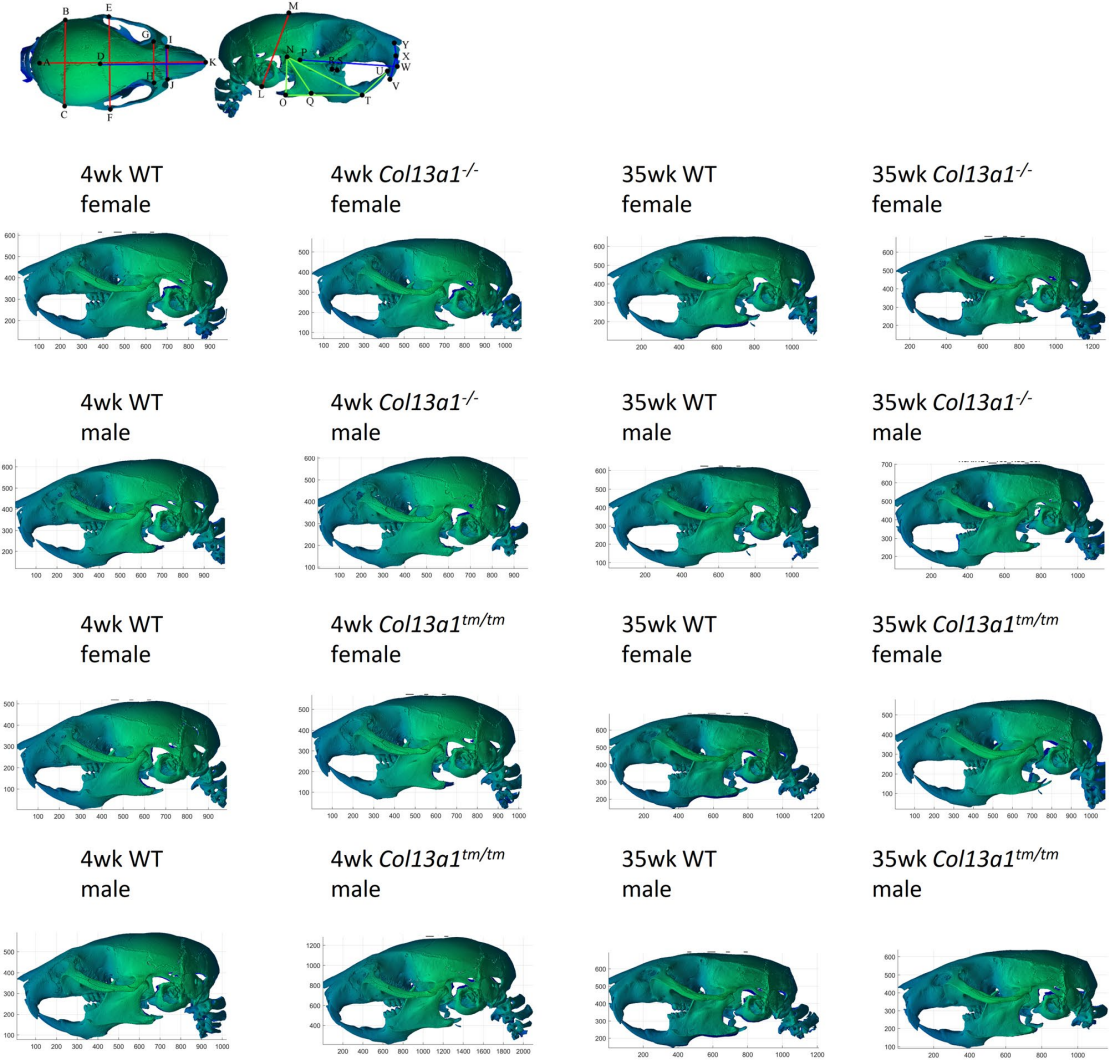
A 3D-reconstructed image of male and female mouse skulls, showing 25 anatomic landmarks used in the cephalometric analysis. Representative images of *Col13a1*^*−/−*^ and *Col13a1*^*tm/tm*^ female mice and respective WT controls at 4- and 35-week time points. For measurement data, see the Supplementary Tables S2 and S2, and Supplementary Figs. S7 and S8.

To evaluate the *Col13a1*^*−/−*^ and the *Col13a1*^*tm/tm*^ mice for possible craniofacial defects, 4- and 35-week-old mouse skulls were imaged using µCT. Single axial and sagittal 2D projection images were generated from the 3D-reconstructed image datasets. From these projection images (Fig. 7), the morphometric analysis (Supplementary Table S1 and Supplementary Figs. S3–S5) was conducted according to a previously published method^24^. In the 4-week-old male and female *Col13a1*^*−/−*^ mice, the interzygomatic length, effective mandibular length and ascending ramus length were reduced compared to the respective WT controls (Supplementary Table S2, Supplementary Figs. S7 and S8). Additionally, the internasal distance was reduced in male, and the upper incisor height, mandibular plane and posterior mandibular height were reduced in female *Col13a1*^*−/−*^ mice compared to the respective WT controls. In the 35-week-old male *Col13a1*^*−/−*^ mice, no significant differences were found in the parameters altered at the 4-week time point. Instead, the interorbitary length was reduced and the inferior incisor length was slightly increased compared to the respective WT controls. In the 35-week-old female *Col13a1*^*−/−*^ mice, the interzygomatic length, mandibular plane and posterior mandibular height were normalized from the 4-week to the 35-week time point, whereas the upper incisor length, mandibular plane and ascending ramus length remained reduced compared to WT (Supplementary Table S2, Supplementary Figs. S9 and S10). In the 4-week-old male *Col13a1*^*tm/tm*^ mice, the upper incisor length and the posterior mandibular height were reduced compared to the WT controls (Supplementary Table S3). At the 35-week time point, the interzygomatic length, palatine length, upper incisor length, inter-molar maxillary distance, ascending ramus length and posterior mandibular height were increased in the male *Col13a1*^*tm/tm*^ mice compared to WT control mice. No significant differences were found in the female *Col13a1*^*tm/tm*^ mice at the 4- or 35-week time points compared to the respective WT controls (Supplementary Table S3).

## Discussion

In this study, we show that the femoral cortical bone area and cortical thickness of *Col13a1*^*−/−*^ and *Col13a1*^*tm/tm*^ mice are reduced in respect to control mice. ColXIII is a membrane-associated collagen existing as transmembrane and shed forms. Previous studies suggest that these two forms have distinct physiological functions^23,25,26^. The role of ColXIII in bone homeostasis has been studied in a transgenic overexpression mouse model (*Col13a1*^*oe*^)^8,12,27^. In the *Col13a1*^*oe*^ mice, excess ColXIII causes a gradual increase of long and calvarial bone mass^27^, disrupts osteoblast differentiation, increases the expression of VEGF in primary osteoblasts and stimulates vascular outgrowth in cultured fetal mouse metatarsals^12^. The pronounced diaphyseal appositional bone mass phenotype is accompanied by endocortical bone loss of similar magnitude^12^. The excess diaphyseal apposition of bone tissue is reduced in ColXIII-overexpressing mice with the simultaneous lack of integrin α11 subunit, demonstrating the in vivo significance of ColXIII-induced signaling in bone turnover^8^.

The role and function of ColXIII in the normal development of postnatal NMJs in *Col13a1*^*−/−*^ and *Col13a1*^*tm/tm*^ mice is well characterized^13,15,23,28^. The muscle tissue of *Col13a1*^*−/−*^ mice has reduced complexity and size of acetylcholine receptor (AChR) clusters already at the age of 4 weeks. In contrast to the *Col13a1*^*−/−*^ mice, the 12-week-old *Col13a1*^*tm/tm*^ mice have increased size and complexity of the AChR clusters compared to WT mice^23^. Here, we show that the femoral bone area and cortical thickness are reduced in both the *Col13a1*^*−/−*^ and the *Col13a1*^*tm/tm*^ mice with aging, long after the occurrence of NMJ abnormalities. In contrast to opposing NMJ phenotypes of *Col13a1*^*−/−*^ and *Col13a1*^*tm/tm*^ mice, the reduction in femoral bone mass was similar in both mouse lines indicating that the reduction of bone mass is not secondary to the abnormal NMJ development, but rather due to a distinct role of ColXIII in bone tissue. We point out that according to marker gene expression bone^13,27^ is in addition to NMJs one of the two tissues with highest ColXIII expression, and thus it is conceivable that the observed bone effects are direct causes of lack of this collagen. However, these data do not exclude the possibility of systemic effects caused by the lack of ColXIII. Additionally, we demonstrate here that the lack of soluble ColXIII in *Col13a1*^*tm/tm*^ mice causes a similar bone phenotype as the total knockout of *Col13a1*, suggesting the importance of soluble ColXIII in bone homeostasis. Previously, we have shown that ColXIII is mainly expressed by diaphyseal osteoblasts in the mouse femur^27^. This could be the cause of the predominant phenotypes in the diaphyseal area in all ColXIII mouse lines, as shown here for *Col13a1*^*−/−*^, *Col13a1*^*tm/tm*^, and previously for *Col13a1*^*oe*^. While the alterations of the cortical metaphysis are not identical with those from the diaphysis, our results are consistent in the sense that cortical bone decreases with age in both anatomical locations. This is in line with prior research, which found that age-related cortical thinning differ between the metaphysis and diaphysis^29^. Previously we have shown that the soluble ColXIII, not the transmembrane form, activates JNK and ERK pathways in osteoblasts^12^. This finding is in accordance with lack of shedding having an impaired effect in the bone formation of the *Col13a1*^*tm/tm*^ mice as shown here. However, the changes in the *Col13a1*^*−/−*^ and the *Col13a1*^*tm/tm*^ mice are similar but not identical. The 72-week-old *Col13a1*^*−/−*^ mice have significantly lowered cortical and medullar areas but not a reduced cortical thickness whereas the *Col13a1*^*tm/tm*^ have a decreased cortical area and cortical thickness but an unaltered medullar area. Age-dependent cortical thinning in mice occurs when the loss of bone from the endosteal surface surpasses the quantity of bone deposited to the periosteal surface^29^. Previous studies demonstrate that this cortical thinning in mice is due to unbalanced endocortical a basic multicellular unit (BMU)-based remodeling, not osteoclastic modeling^29^. Based on our previous findings, soluble ColXIII affects specifically BMU-based remodeling^12^. The decrease observed here in the medullar expansion of the old *Col13a1*^*−/−*^ mice is consistent with these previous results, indicating the importance of ColXIII in BMU-based cortical remodeling. However, based on the data presented here, we cannot explain why in the *Col13a1*^*tm/tm*^ mice cortical thinning is increased without changes in the medullary expansion compared to WT. Nevertheless, the phenotypic similarity in bone of the *Col13a1*^*−/−*^ and *Col13a1*^*tm/tm*^ mice suggests that the soluble ColXIII is the main form affecting bone homeostasis, but also the transmembrane form appears to have some direct or indirect roles in bone remodeling.

Similarly to CMS19 patients^17,19^, the *Col13a1*^*−/−*^ and the *Col13a1*^*tm/tm*^ mice exhibit visible craniofacial malformations. However, the cephalometric analysis of the *Col13a1*^*−/−*^ and the *Col13a1*^*tm/tm*^ mice did not reveal a clear phenotypical pattern when both sexes were studied at two time points, though it revealed subtle and sex-dependent alterations whose origin remain unclear. Moreover, some differences found in the 4 weeks old mice became nonsignificant at the 35-week time point, possibly because of delayed and abnormal postnatal development being overcome with age. Alterations of craniofacial morphology have been published in several mouse models of syndromic craniosynostoses. Whereas clear craniofacial abnormalities have been reported for example in mouse lines with mutations of *FGFR1*^30^, *FGFR2*^31–34^, *TCF12*^35^ and *EFNB1*^36^, several mutant mouse models have been reported to present with only mild calvarial abnormalities compared to the respective human patients^37–42^. These mouse lines, however, often present with other skeletal abnormalities such as decreased femoral cortical thickness and cortical bone area^41–43^ or decreased vertebral BV/TV and BMD^44^.

In conclusion, our findings in the skeletal tissues show that lack of ColXIII in mice indeed affects bone, although the findings are less pronounced than in the human disease, which is often the case also in other mouse models of human syndromes. Unlike the NMJ alterations, which stabilize with age in the *Col13a1*^*−/−*^ mice and the CMS19 patients^17,28^, the reduced bone phenotype becomes aggravated with aging of the mice. This finding may be significant also in understanding the effects of lack of ColXIII in humans. As such, our results further validate and expand the potential use of the *Col13a1*^*−/−*^ mice as a disease model for CMS19. Although the abnormal development of NMJs shows marked differences between the *Col13a1*^*−/−*^ and the *Col13a1*^*tm/tm*^ mice, we found the tubular bone mass to be similarly reduced in both mouse lines. This would indicate that especially soluble ColXIII has a significant role in the upkeep of cortical bone mass with aging, independent of its functions in normal NMJ development. As both complete and partial lack of ColXIII reduced femoral bone mass significantly, future research is warranted to assess the possibility of bone pathologies such as osteoporosis in human CMS19 patients.

## Material and methods

### Mouse models

Generation of *Col13a1*^*−/−*^ (MGI:4838409) and *Col13a1*^*tm/tm*^ (MGI:6116692) mice has been described previously in their respective publications^13,23^. *Col13a1*^*−/−*^ as a CMS19 model has been described previously^23^.

### Low-resolution micro-computed tomography (µCT)

For µCT analyses, left femurs were dissected from male and female *Col13a1*^*tm/tm*^ and *Col13a1*^*−/−*^ mice at 4-, 12-, 25-, 35- and 72-week time points. Samples were also collected from age and gender matched WT littermate controls. Samples were stored in phosphate-buffered saline at -20 °C with no prior fixation. Most recent guidelines for µCT methodology were used^45^. Samples were imaged with a Skyscan 1176 scanner (Bruker microCT, Kontich, Belgium) designed for in vivo imaging of live animals. X-ray tube voltage of 50 kV with image pixel size of 8.71 μm and current of 500 µA with a 0.5 mm aluminum filter were used. One image projection was collected every 0.3° over 360° rotation with an exposure time of 4000 ms. Specimens were imaged without a scanning medium (i.e. air) wrapped in a moist paper and placed in a sealed container to prevent drying. Image slices were reconstructed into the tomographic image datasets using NRecon software (Skyscan, v. 1.6.5.2), where ring artefact and beam hardening corrections were applied. 2D and 3D volumetric analyses of datasets were done using CTAn software (Skyscan, v. 1.11.10.0). Both softwares were provided by the manufacturer. Regions of interest (ROIs) were manually drawn for trabecular and cortical bone quantitative morphometric analyses. Trabecular bone was assessed from distal femoral metaphyseal area using an irregular, anatomic region of interest drawn manually a few voxels away from the endocortical surface. Trabecular ROI continued 0.9 mm proximally starting from the distal growth plate. Following 3D morphometric parameters were measured: total volume of region of interest (TV), volume of the region segmented as bone (BV), bone volume fraction (BV/TV) and trabecular number (Tb.N). Diaphyseal cortical bone was assessed using a volume of interest starting 0.9 mm from femoral neck and extending distally towards the diaphysis. The volume of interest (VOI) enclosed the whole sample from the assessed section. Due to normal longitudinal growth, two different VOI lengths were used. For 4-week time point, a length of 3.6 mm (400 axial slices) was measured. For 12, 25, 35 and 72-week time points, a length of 5.4 mm (600 axial slices) was measured. Following 2D morphometric parameters were measured: cortical bone area (Ct.Ar), medullary area (Ma.Ar) and average cortical thickness (Ct.Th). For data visualization and femur length measurements, DataViewer software (v. 1.5.6.2) was used. Femur length was measured from the top of trochanter major in mid frontal plane to the distal patellofemoral groove. Measurements were repeated three times and the mean value was used as the result.

### High-resolution µCT

For more detailed analysis of bone morphometric parameters, left mouse femurs were analyzed from *Col13a1*^−/−^ and *Col13a1*^*t*m/tm^ female mice using high-resolution computed tomography (HRCT). Samples from the 35- and 72-week time points were analyzed, and age-matched WT control samples were used. Skyscan 1272 (Bruker microCT, Kontich, Belgium) scanner, designed for high-resolution 3D imaging of ex vivo specimens, were used with an X-ray tube voltage of 50 kV, a current of 200 μA, an exposure time of 4000 ms and a 0.25-mm aluminum filter. X-ray images were acquired every 0.29° within a 360° rotation. Final image resolution was 1.75 μm. A sealed container and moist tissue paper were used to prevent sample drying during imaging. The image datasets were processed, and image artifact corrections were applied using NRecon software (v. 1.6.5.2). The datasets were reoriented using the Dataviewer software (v. 1.5.6.2) and morphometric analyses were conducted using the CTAn software (v. 1.11.10.0). All softwares were provided by the manufacturer. The reporting and discussion of the results followed current recommendations^45,46^. Trabecular bone was assessed from the distal femoral metaphyseal area using an anatomic ROI drawn a few voxels away from the bone endocortical surface. The length of the trabecular ROI was 0.7 mm (400 axial slices) starting from the distal growth plate and extending towards the diaphysis. Following 3D morphometric parameters were measured: TV, BV, BV/TV and Tb.N. Metaphyseal cortical bone was assessed using a 0.35-mm-long (200 axial slices) VOI starting 1.75 mm from the distal growth plate and extending proximally towards the diaphysis. Following 2D morphometric parameters were measured: Ct.Ar, Ma.Ar, and Ct.Th.

### Cephalometric analysis

For cephalometric analyses, we imaged *Col13a1*^*−/−*^ and *Col13a1*^*tm/tm*^ mouse skulls at 4- and 35-week time points using HRCT. Both genders were studied, and age- and gender-matched WT littermate controls were used. The samples were stored at − 20 °C in phosphate-buffered saline (PBS) without prior fixation. The samples were imaged using a Skyscan 1272 (Bruker microCT, Kontich, Belgium) scanner with a tube voltage of 50 kV and 0.5 mm aluminum filter. X-ray images were collected every 0.29° within a 360° rotation with an exposure time of 1550 ms. The final image resolution was 10.7 μm. The samples were covered in moist tissue paper in a sealed container during imaging to prevent drying. Image processing was done using the NRecon software (v. 1.6.5.2) and the datasets were reoriented using the DataViewer software (v. 1.5.6.2). Softwares were provided by the manufacturer. An in-house MATLAB (R2018b, The MathWorks, USA) script was used to perform the cephalometric analyses (Supplementary material and Supplementary Figs. S4–S6). A sagittal and transverse projection image of 3D reconstructed samples was produced from each dataset and saved with a corresponding filename. Measurements for craniofacial, maxillary, and mandibular morphology were conducted similarly to a previously published list of measurements^24^. The samples were measured in a randomized order and the filenames were hidden from the user during measurement to prevent bias. All measurements were performed three times, and the mean value was used as the result.

### Statistics

Graphical data are shown either in boxplots representing data values from 25 to 75th percentile or in bar charts. In boxplots, the line indicates the median value and the whiskers represent the complete spread of all samples in the group (min to max). In the bar charts, the whiskers represent the range of the dataset. The data were not tested for normality. Data were analyzed with two-way analysis of variance (ANOVA) using the GraphPad Prism software (GraphPad Software, v. 7.03). Multiple comparisons were performed between the groups at the respective time points. P-values were adjusted using the Benjamini, Krieger and Yekutieli false discovery rate (q)^47^. Finally, q < 0.05 was considered a statistically significant result.

### Study approval

All applicable international, national, and institutional guidelines for the care and use of animals were followed and all experimental protocols were approved by the Animal Use and Care Committee of the University of Oulu and the National Animal Experiment Board (Eläinkoelautakunta ELLA). All procedures performed in studies involving animals were in accordance with the ethical standards of the European Community Council Directive on the protection of animals used for scientific purposes (September 22, 2010; 2010/63/EEC), national legislation and the regulations for the care and use of laboratory animals. This study was carried out in compliance with the ARRIVE guidelines.

## Supplementary Information

Supplementary Information.
