# Supplementary material for: The CMS19 disease model specifies a pivotal role for collagen XIII in bone homeostasis
Source: Sci Rep. 2022 Apr 7;12:5866. doi: 10.1038/s41598-022-09653-4 (PMC8990013; doi:10.1038/s41598-022-09653-4)
Supplement: Supplementary file 1 — Supplementary Information. [file 41598_2022_9653_MOESM1_ESM.pdf]

The CMS19 disease model specifies a pivotal role for collagen XIII in bone homeostasis

Kemppainen AV[a], Finnilä MA[b], Heikkinen A[a], Härönen H[a], Izzi V[a,c,d], Kauppinen S[b], Saarakkala S[b,e], Pihlajaniemi T[a], Koivunen J[a,*]

[a]ECM-Hypoxia research unit, Faculty of Biochemistry and Molecular Medicine, University of Oulu, P.O. Box 5400, Oulu, FIN-90014, Finland

[b]Research Unit of Medical Imaging, Physics and Technology, University of Oulu, P.O. Box 5000, Oulu, FIN-90014, Finland

[c]Faculty of Medicine, University of Oulu, Oulu, FI-90014, Finland

[d]Finnish Cancer Institute, Helsinki, 00130, Finland

[e]Department of Diagnostic Radiology, Oulu University Hospital, Oulu, Finland

*Correspondence: jarkko.koivunen@oulu.fi

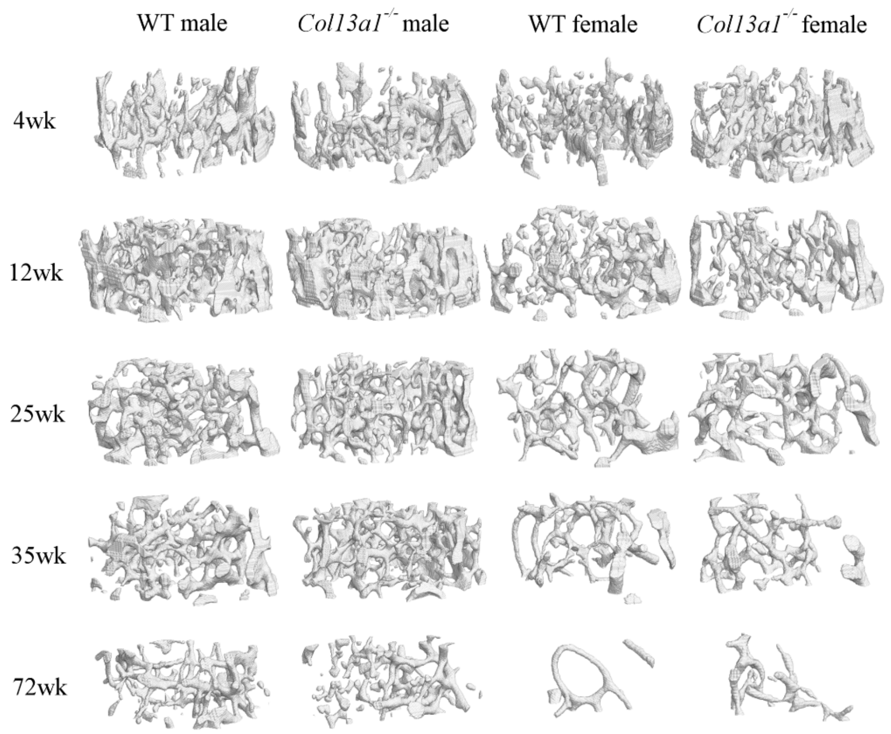

Fig S1. Representative 3D reconstruction images of distal femoral trabecular bone in Col13a1$^{-/-}$ male and female mice and respective controls. The trabecular architecture and the bone mass were similar between genotypes at all time points.

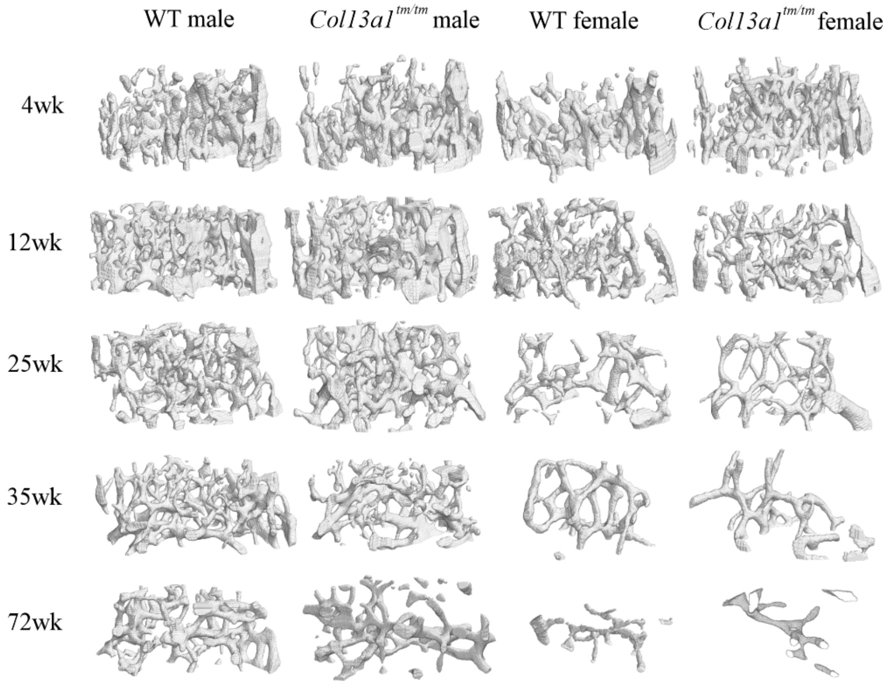

Fig S2. Representative 3D reconstruction images of distal femoral trabecular bone in male and female Col13a1$^{tm/tm}$ mice and respective WT controls. Visual inspection of metaphyseal trabecular bone did not show clear differences between the Col13a1$^{tm/tm}$ and WT samples in either gender at any time point studied.

A

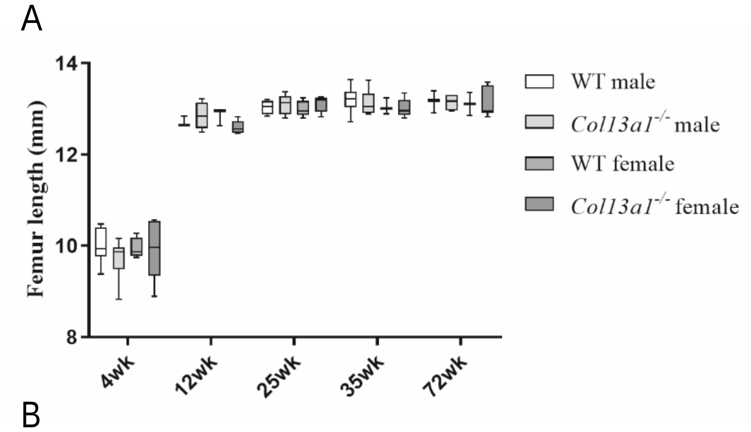

B

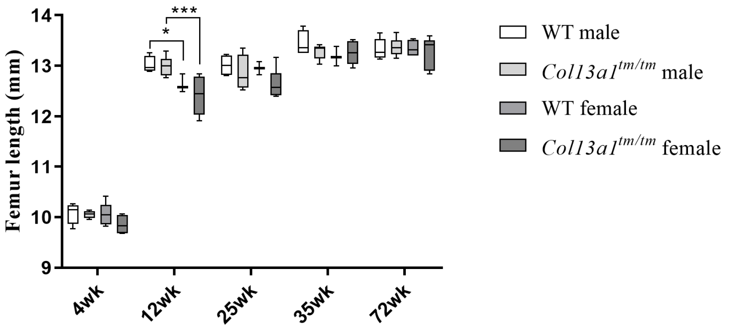

Fig S3. Femoral length of the Col13a1$^{-/-}$ (A) and Col13a1$^{tm/tm}$ (B) male and female mice and respective WT controls. The femoral length was unaltered in the Col13a1$^{-/-}$ and Col13a1$^{tm/tm}$ mice compared to the WT controls. n(WT male): 3-6; n(Col13a1$^{-/-}$ male): 4-7; n(WT female): 3-6; n(Col13a1$^{-/-}$ female): 5-6; n(WT male): 4-6; n(Col13a1$^{tm/tm}$ male): 4-6; n(WT female): 2-6; n(Col13a1$^{tm/tm}$ female): 4-6; the whiskers represent the total range of the dataset (min to max).

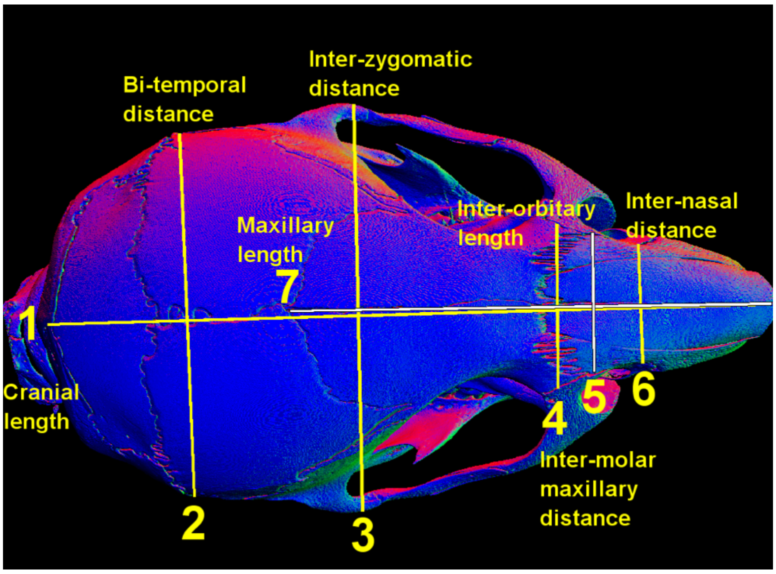

Fig S4. The top view image and its measurements.

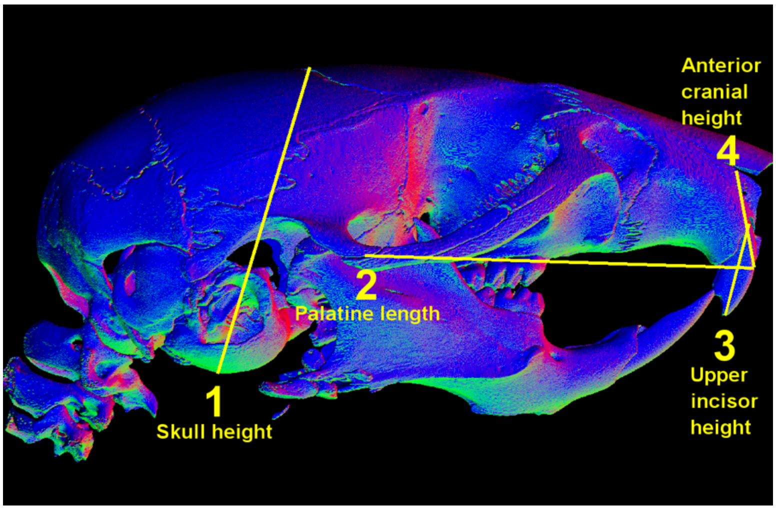

Fig S5. The first side view image and its measurements.

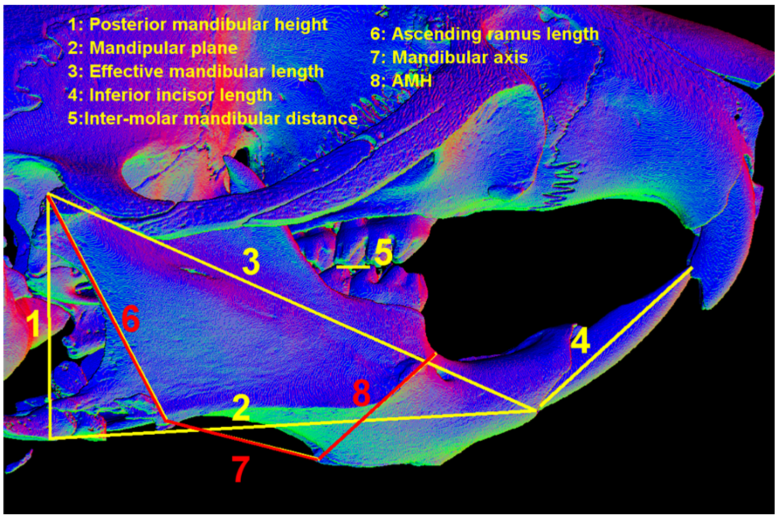

Fig S6. The second side view image and its measurements.

Table S1. List of morphometric parameters and their definitions as they are used in this study. Morphometric parameters are defined here as measured distances between two anatomic points in 2D sagittal and transverse projection images. Morphometric parameters are divided into three categories (craniofacial, maxillary and mandibular) based on their anatomic localization. Modified from Eimar et al. 2016.

Craniofacial		Maxillary		Mandibular	
Cranial length	A-K	Maxillary length	D-K	Effective mandibular length	N-T
Skull height	L-M	Palatine length	P-W	Mandibular plane	O-T
Internasal distance	I-J	Anterior cranial height	Y-W	Inferior Incisor length	T-U
Interorbital length	G-H	Upper incisor length	V-X	Ascending ramus length	N-Q
Interzygomatic length	E-F	Intermolar maxillary distance	I-J	Posterior mandibular height	N-O
Bi-temporal distance	B-C			Intermolar mandibular distance	R-S

Table S2. Cephalometric analysis of the male and female Col13a1$^{-/-}$ mice and respective controls at the ages of 4 and 35 weeks. The mean is presented as the result for each group. Several parameters were significantly altered in the Col13a1$^{-/-}$ samples at both time points. n(male): 4-5; n(female): 4-5; *, q<0.05; **, q<0.01; ***, q<0.001 determined by two-way ANOVA and followed by the false discovery rate.

	4 wk					35 wk				
Male	+/+ (mm)	SD	-/-	SD	q	+/+	SD	-/-	SD	q
Cranial length	19,77	0,74	19,57	0,63	0,236	22,89	0,69	22,53	0,23	0,188
Skull height	9,09	0,17	9,13	0,20	0,815	9,17	0,15	9,38	0,12	0,227
Internasal distance	3,48	0,08	**3,38***	0,02	0,035	3,74	0,08	3,73	0,05	0,250
Interorbital length	5,12	0,10	5,15	0,04	0,222	5,53	0,10	**5,41***	0,07	0,039
Interzygomatic length	11,41	0,26	**11,08***	0,24	0,030	12,49	0,23	12,55	0,09	0,249
Bi-temporal distance	10,23	0,14	10,21	0,10	0,305	10,55	0,10	10,48	0,14	0,188
Maxillary length	13,23	0,44	13,07	0,51	0,231	16,26	0,56	15,74	0,19	0,058
Palatine length	11,29	0,14	10,94	0,42	0,055	13,55	0,30	13,54	0,14	0,330
Anterior cranial height	2,34	0,02	2,34	0,08	0,339	2,81	0,07	2,78	0,05	0,260
Upper incisor length	3,01	0,08	2,92	0,14	0,133	3,55	0,14	3,54	0,07	0,344
Intermolar maxillary distance	4,12	0,12	4,20	0,05	0,105	4,43	0,08	4,39	0,05	0,190
Effective mandibular length	9,59	0,06	**9,11****	0,27	0,002	11,35	0,27	11,35	0,13	0,173
Mandibular plane	7,15	0,24	6,93	0,21	0,092	7,95	0,25	7,90	0,16	0,277
Inferior incisor length	3,98	0,10	3,96	0,11	0,268	4,74	0,13	**4,89***	0,12	0,043
Ascending ramus length	4,79	0,09	**4,68***	0,22	0,046	5,80	0,19	5,79	0,11	0,311
Posterior mandibular height	4,74	0,10	4,61	0,20	0,096	5,39	0,11	5,55	0,13	0,072
Inter-molar mandibular distance	0,55	0,01	0,58	0,04	0,819	0,56	0,04	0,54	0,04	0,852
Female	+/+ (mm)	SD	-/-	SD	q	+/+	SD	-/-	SD	q
Cranial length	19,79	0,68	19,25	0,91	0,134	22,30	0,33	22,07	0,56	0,229
Skull height	9,09	0,21	8,89	0,18	0,343	9,21	0,14	9,26	0,15	0,731
Iinternasal distance	3,46	0,08	3,35	0,10	0,282	3,58	0,06	3,55	0,12	0,730
Interorbital length	5,04	0,06	5,02	0,10	0,226	5,22	0,02	5,30	0,07	0,064
Interzygomatic length	11,35	0,12	**10,86****	0,36	0,004	12,09	0,11	12,09	0,22	0,174
Bi-temporal distance	10,28	0,04	10,12	0,11	0,138	10,44	0,15	10,38	0,13	0,394
Maxillary length	13,29	0,61	12,71	0,85	0,102	15,74	0,33	15,41	0,41	0,166
Palatine length	11,39	0,21	10,97	0,58	0,063	13,43	0,11	13,13	0,30	0,094
Anterior cranial height	2,40	0,05	2,37	0,02	0,216	2,73	0,10	2,68	0,05	0,130
Upper incisor length	3,07	0,08	**2,94***	0,04	0,023	3,51	0,11	**3,33****	0,15	0,007
Intermolar maxillary distance	4,17	0,07	4,07	0,10	0,402	4,19	0,09	4,26	0,10	0,462
Effective mandibular length	9,75	0,23	**9,17****	0,35	0,001	11,20	0,12	**10,91***	0,13	0,020
Mandibular plane	7,31	0,26	**6,81***	0,29	0,013	7,46	0,07	7,57	0,16	0,253
Inferior incisor length	3,95	0,15	3,89	0,22	0,247	4,91	0,10	4,85	0,17	0,247
Ascending ramus length	4,86	0,30	**4,55***	0,25	0,033	6,01	0,09	**5,64***	0,15	0,018
Posterior mandibular height	4,75	0,28	**4,45***	0,26	0,039	5,47	0,07	5,25	0,13	0,062
Inter-molar mandibular distance	0,56	0,02	0,51	0,02	0,064	0,58	0,04	0,54	0,03	0,129

Table S3. Cephalometric analysis of the male and female Col13a1$^{tm/tm}$ mice and respective controls at the ages of 4 and 35 weeks. The mean is presented as the result for each group. Statistically significant differences were mostly found in the 35-week-old Col13a1$^{tm/tm}$ male samples, where several parameters were increased. There were no statistically significant differences between the female Col13a1$^{tm/tm}$ mice and the respective controls at either time point. n(male): 3-6; n(female): 3-4. *, $q<0.05$; **, $q<0.01$; ***, $q<0.001$ determined by two-way ANOVA and followed by the false discovery rate.

	4 wk					35 wk				
Male	+/+	SD	tm/tm	SD	q	+/+	SD	tm/tm	SD	q
	(mm)									
Cranial length	20,09	0,30	20,22	0,12	0,199	21,78	0,63	22,13	0,25	0,103
Skull height	9,04	0,26	9,08	0,11	0,870	9,29	0,09	9,11	0,11	0,470
Iinternasal distance	3,41	0,07	3,40	0,03	0,306	3,70	0,11	3,76	0,02	0,109
Interorbital length	5,10	0,11	5,13	0,09	0,422	5,27	0,13	5,43	0,05	0,070
Interzygomatic length	11,36	0,12	11,38	0,09	0,311	12,23	0,36	**12,53***	0,09	0,026
Bi-temporal distance	10,30	0,02	10,28	0,08	0,756	10,35	0,06	10,44	0,16	0,550
Maxillary length	13,30	0,33	13,42	0,24	0,214	15,25	0,56	15,62	0,16	0,088
Palatine length	11,34	0,15	11,39	0,16	0,138	12,89	0,59	**13,47****	0,09	0,006
Anterior cranial height	2,40	0,08	2,41	0,03	0,302	2,72	0,10	2,86*	0,07	0,018
Upper incisor length	3,06	0,09	**2,97***	0,07	0,035*	3,39	0,13	**3,59****	0,11	0,007
Intermolar maxillary distance	4,16	0,05	4,10	0,04	0,078	4,36	0,10	**4,45***	0,07	0,043
Effective mandibular length	9,68	0,2	9,68	0,07	0,349	10,90	0,45	11,04	0,11	0,206
Mandibular plane	7,15	0,15	7,17	0,28	0,318	7,90	0,18	7,84	0,21	0,318
Inferior incisor length	3,99	0,07	4,08	0,06	0,147	4,71	0,25	4,93	0,20	0,051
Ascending ramus length	4,89	0,06	4,82	0,07	0,079	5,55	0,27	**5,86****	0,12	0,004
Posterior mandibular height	4,86	0,04	**4,63***	0,19	0,019	5,28	0,21	**5,51***	0,12	0,035
Inter-molar mandibular distance	0,55	0,05	0,56	0,02	0,947	0,59	0,01	0,58	0,03	0,879
Female	+/+	SD	tm/tm	SD	q	+/+	SD	tm/tm	SD	q
	(mm)									
Cranial length	20,00	0,45	19,94	0,33	0,288	22,35	0,30	22,22	0,30	0,286
Skull height	9,10	0,10	9,08	0,08	0,930	9,27	0,13	9,18	0,13	0,494
Iinternasal distance	3,38	0,04	3,40	0,08	0,212	3,70	0,04	3,76	0,04	0,091
Interorbital length	5,05	0,07	5,08	0,18	0,550	5,29	0,09	5,36	0,09	0,426
Interzygomatic length	11,29	0,16	11,37	0,28	0,214	12,24	0,09	12,15	0,09	0,214
Bi-temporal distance	10,23	0,12	10,18	0,08	0,685	10,38	0,10	10,36	0,10	0,893
Maxillary length	13,47	0,45	13,27	0,22	0,154	15,60	0,17	15,20	0,17	0,067
Palatine length	11,20	0,26	11,12	0,31	0,223	13,62	0,07	13,34	0,07	0,069
Anterior cranial height	2,36	0,03	2,41	0,11	0,190	2,71	0,12	2,73	0,12	0,291
Upper incisor length	2,95	0,11	3,02	0,07	0,173	3,39	0,13	3,59	0,13	0,087
Intermolar maxillary distance	4,15	0,04	4,11	0,10	0,158	3,69	0,04	3,62	0,04	0,113
Effective mandibular length	9,64	0,26	9,54	0,18	0,249	11,25	0,18	11,24	0,18	0,332
Mandibular plane	7,30	0,25	7,08	0,31	0,381	7,57	0,05	7,38	0,05	0,425
Inferior incisor length	3,91	0,17	4,08	0,18	0,076	5,03	0,10	4,99	0,10	0,272
Ascending ramus length	4,87	0,11	4,80	0,15	0,201	6,00	0,13	6,07	0,13	0,201
Posterior mandibular height	4,87	0,17	4,71	0,12	0,069	5,50	0,10	5,49	0,10	0,318
Inter-molar mandibular distance	0,54	0,03	0,57	0,01	0,445	0,59	0,03	0,56	0,03	0,445

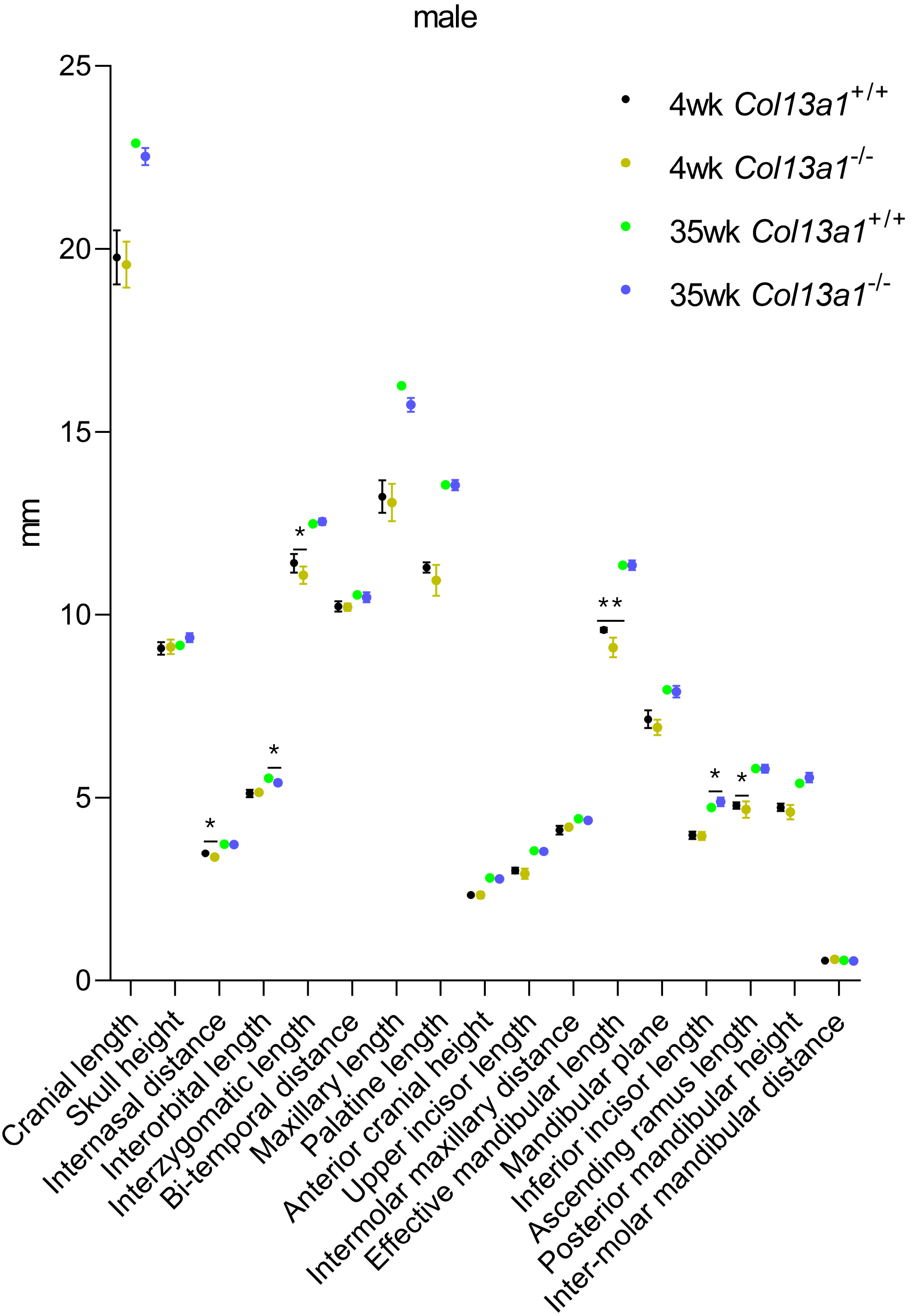

Fig S7. Cephalometric analysis of the male Col13a1$^{-/-}$ mice and respective controls at the ages of 4 and 35 weeks presented as mean±SD. n: 4-5; *, q<0.05; **, q<0.01; determined by two-way ANOVA and followed by the false discovery rate.

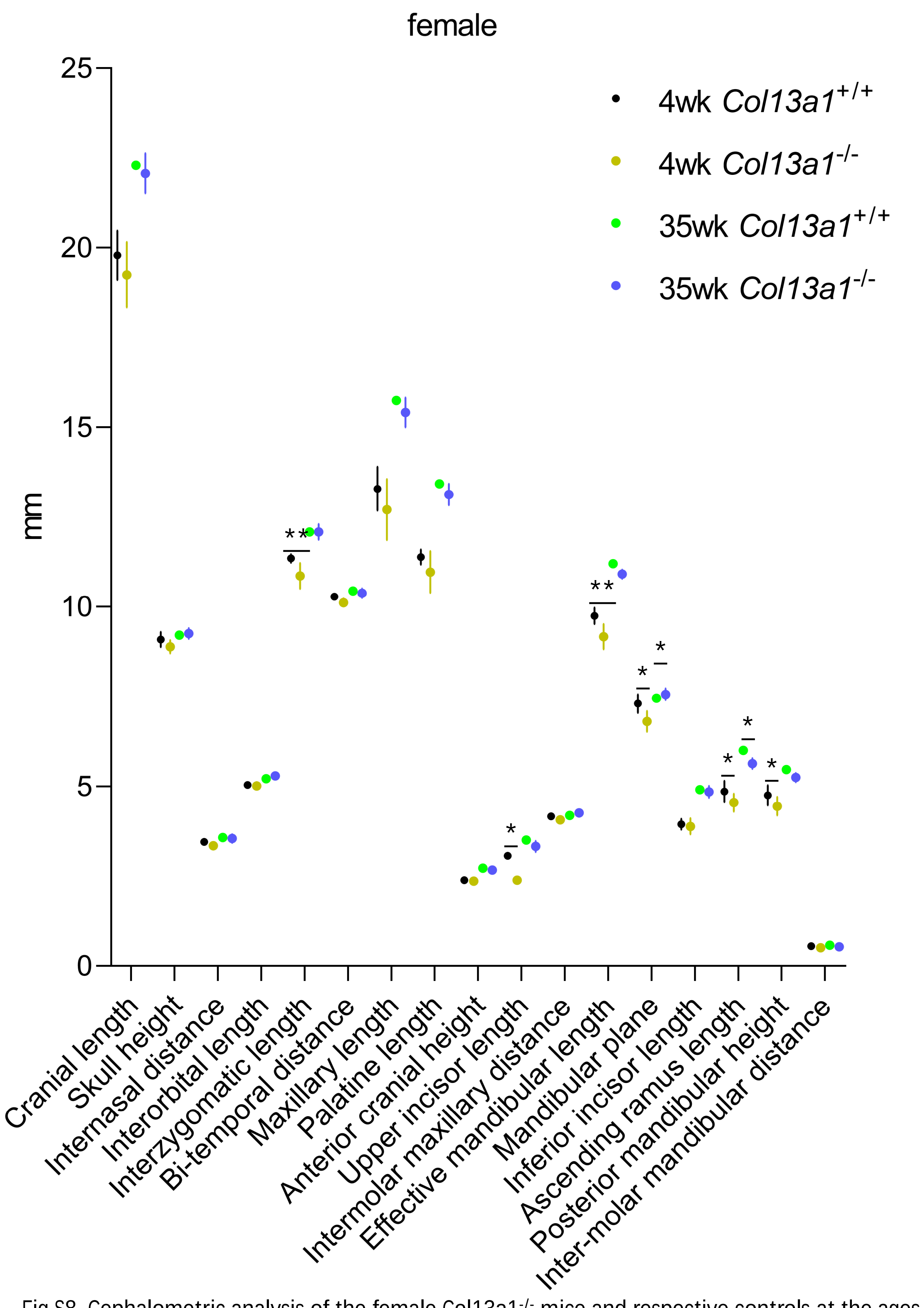

Fig S8. Cephalometric analysis of the female Col13a1$^{-/-}$ mice and respective controls at the ages of 4 and 35 weeks presented as mean±SD. n: 4-5; *, $q<0.05$; **, $q<0.01$; determined by two-way ANOVA and followed by the false discovery rate.

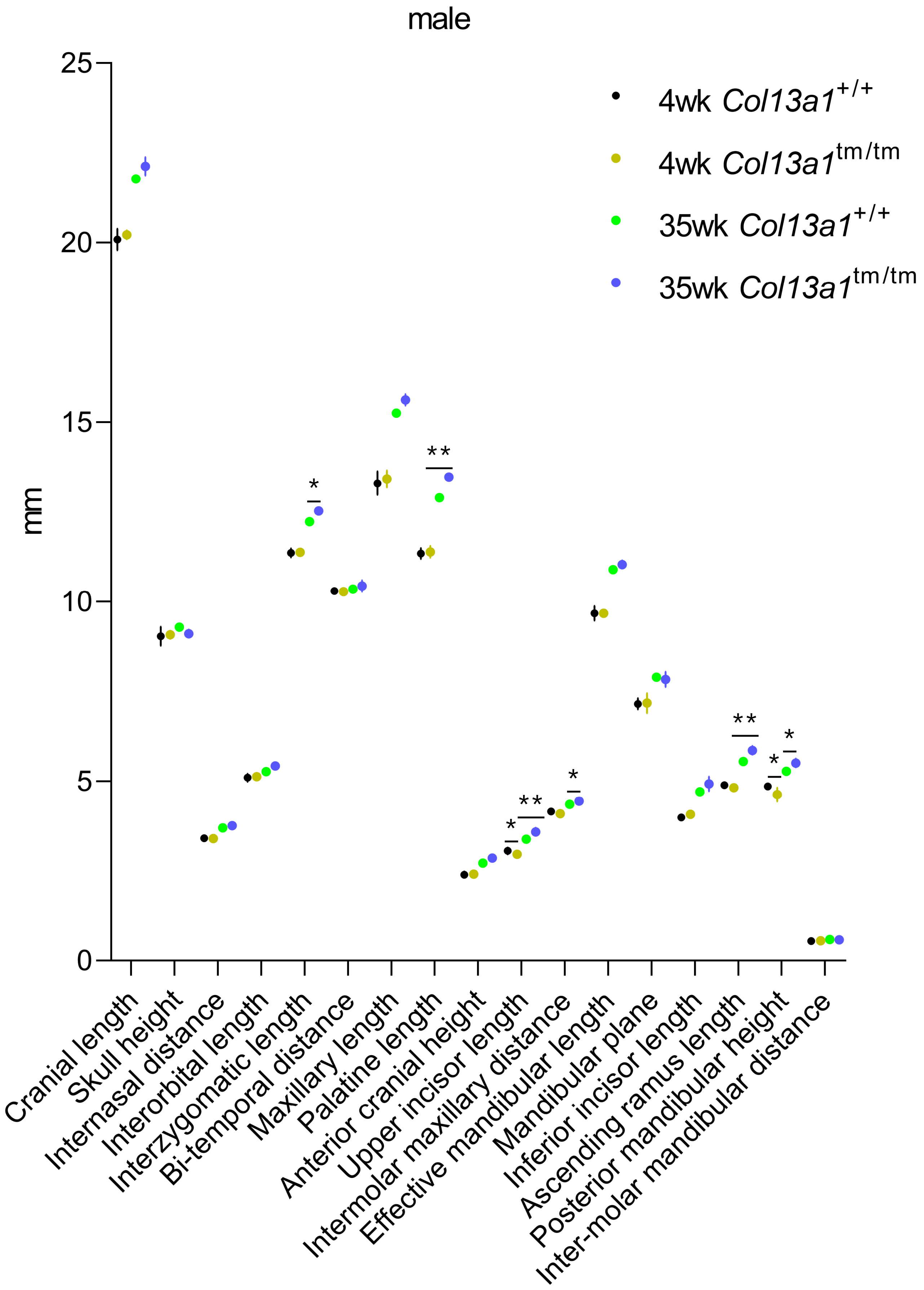

Fig S9. Cephalometric analysis of the male Col13a1$^{tm/tm}$ mice and respective controls at the ages of 4 and 35 weeks presented as mean±SD. n: 3-6; *, q<0.05; **, q<0.01; determined by two-way ANOVA and followed by the false discovery rate.

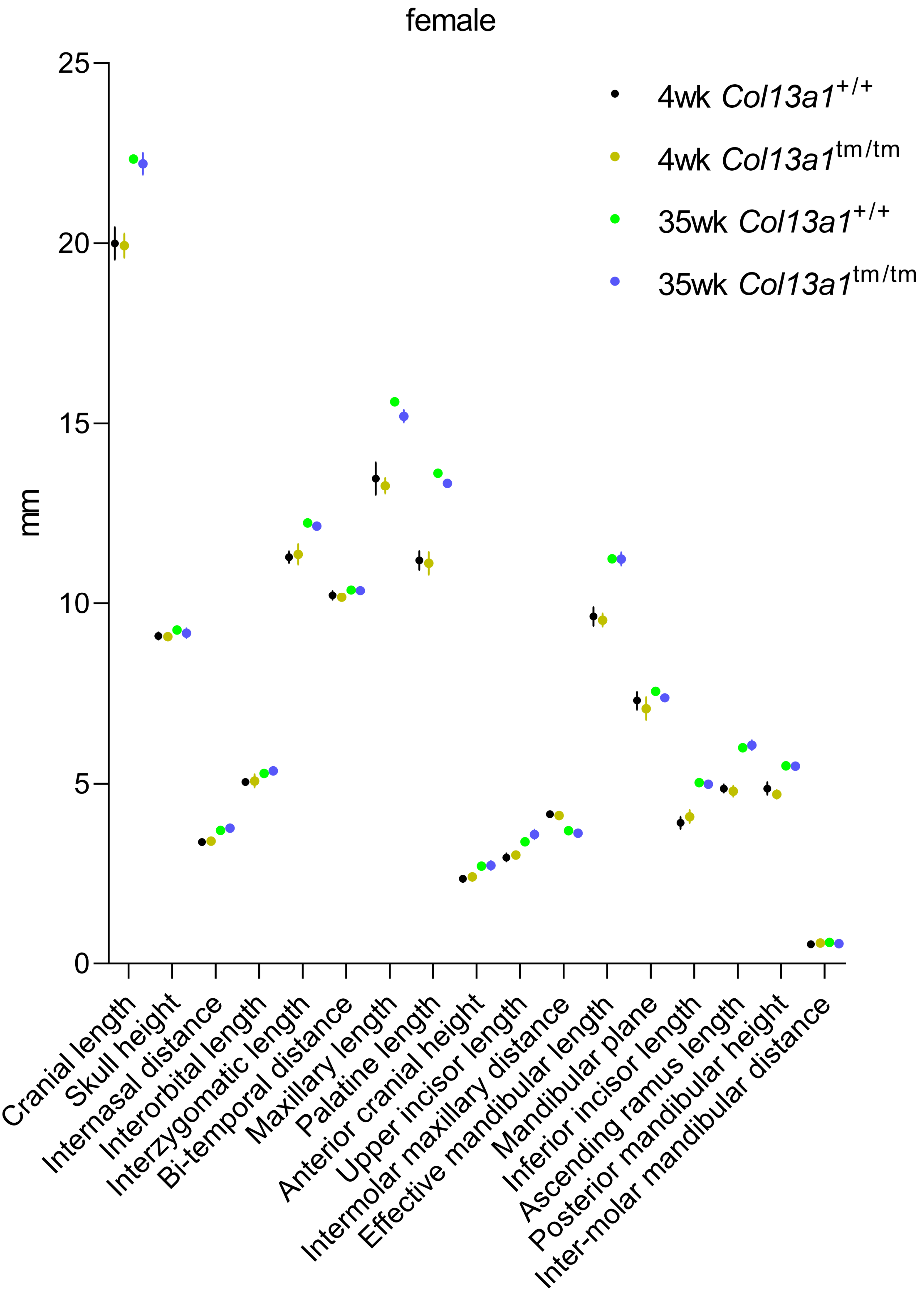

Fig S10. Cephalometric analysis of the female Col13a1$^{tm/tm}$ mice and respective controls at the ages of 4 and 35 weeks presented as mean±SD. No statistical differences were discovered by two-way ANOVA and followed by the false discovery rate.
